# The APC/C subunit APC7 exerts antiviral effects by targeting the adaptor protein MAVS

**DOI:** 10.3389/fimmu.2026.1912055

**Published:** 2026-08-11

**Authors:** Rui Su, Aiping Sun, Yifan Niu, Tiesuo Zhao, Hui Wang

**Affiliations:** 1 Department of Immunology, School of Basic Medical Sciences, Henan Medical University, Xinxiang, China; 2 Xinxiang Engineering Technology Research Center of Immune Checkpoint Drug for Liver-Intestinal Tumors, Henan Medical University, Xinxiang, China; 3 Henan Collaborative Innovation Center of Molecular Diagnosis and Laboratory Medicine, School of Medical Technology, Henan Medical University, Xinxiang, China

**Keywords:** APC7, IFN-I, innate immunity, MAVS, RNA virus

## Abstract

The innate immune response is the first line of host defense against viral infection. RNA virus infection triggers activation of retinoic acid-inducible gene-I (RIG-I)-mitochondrial antiviral signaling protein (MAVS) signaling pathway, resulting in the formation of prion-like aggregates of MAVS and production of type I interferons (IFN-I). Here, we found that APC7, a subunit of the anaphase-promoting complex/cyclosome (APC/C), can significantly restrict the replication of RNA viruses including Enterovirus 71 (EV71) and vesicular stomatitis virus (VSV). Further experiments showed that overexpression of APC7 enhances RNA virus-induced IFN-I expression, whereas knockdown of APC7 reduces it. Moreover, APC7 regulated the innate immune response independently of APC/C catalytic function. Subsequent analysis indicated that APC7 is partly localized to mitochondria, where it interacts with the transmembrane domain of MAVS. Furthermore, we discovered that APC7 promotes K63-linked polyubiquitination and mitochondrial aggregation of MAVS after RNA virus infection. Taken together, these findings demonstrated that APC7 potentiates the antiviral response through activation of MAVS-mediated signaling, which provides new insights into the regulatory mechanisms of innate immunity and viral infections.

## 1 Introduction

The innate immune system recognizes invading pathogens through pattern recognition receptors (PRRs), such as Toll-like receptors (TLRs), cytoplasmic DNA sensors, and retinoic acid-inducible gene I (RIG-I)-like receptors (RLRs) (1, 2). Upon binding to cytoplasmic viral RNA, RIG-I undergoes conformational changes and recruits mitochondrial antiviral signaling protein (MAVS) (3, 4). Activation of MAVS induces the formation of prion-like aggregates on mitochondria (5, 6). Subsequently, MAVS activates TBK1 and IKK kinase complexes, resulting in phosphorylation and nuclear translocation of transcription factors interferon regulatory factor 3 (IRF3) and nuclear factor κB (NF-κB) (7). These transcription factors ultimately promote the production of proinflammatory cytokines and type I interferons (IFN-I), which in turn induce the expression of a series of interferon-stimulated genes (ISGs) that mediate antiviral effects (8).

MAVS serves as a central hub connecting virus recognition and antiviral immunity, and its prion-like aggregates are a hallmark of MAVS activation (5). Posttranslational modifications play an important role in modulating the aggregation and activation of MAVS (9). During viral infection, the E3 ubiquitin ligase TRIM31 promotes the formation of MAVS aggregates through mediating K63-linked ubiquitination (10). The E3 ubiquitin ligases ANKIB1 and pVHL regulate K48-linked ubiquitination of MAVS, which subsequently results in its degradation via the ubiquitin-proteasome pathway (11, 12). Conversely, the counteraction of K48-linked ubiquitination of MAVS by deubiquitinase OTUD4 enhances MAVS-mediated signal transduction (13). Other types of posttranslational modifications, including O-linked N-acetylglucosamine (O-GlcNAc), succinylation, methylation, and palmitoylation, also modulate MAVS activity and aggregation levels (14–17). In addition, various non-modification proteins are involved in regulating MAVS signaling. For example, the endoplasmic reticulum protein ATP13A1 and the mitochondrial-localized protein ALDH1B1 facilitate the aggregation of MAVS and the antiviral immune response (18, 19). In contrast, WDR77 inhibits prion-like aggregation of MAVS and dampens IFN-I signaling (20). Therefore, MAVS aggregate formation is a tightly controlled process in the antiviral response.

The anaphase-promoting complex/cyclosome (APC/C) is a multisubunit E3 ubiquitin ligase that performs an essential function in controlling mitotic progression by binding to coactivators Cdc20 or Cdh1 to promote the degradation of cell cycle regulatory proteins (21–23). APC7 is a core component of APC/C responsible for substrate recruitment (24). Structurally, the N-terminus of APC7 contains a dimerization domain that mediates the formation of APC7 homodimers, while the C-terminus contains multiple tetratricopeptide repeat domains (TPR) that interact with Cdc20 and Cdh1 (24, 25). Although loss of APC7 has little impact on mitotic APC/C function, deletion of APC7 attenuates APC/C ubiquitination activity and allows proliferation of human cells without the spindle assembly checkpoint (26). A recent study demonstrated that APC7 is required for chromatin-associated Ki-67 degradation by APC/C, and humans or mice with APC7 mutation or deletion exhibit intellectual decline and cognitive impairments (27). In addition, *Bifidobacterium infantis* upregulates APC7 expression in colon tissue, thereby enhancing genomic stability and alleviating colonic inflammation (28). However, the effect of APC7 on viral infection and innate immunity has not been defined.

In this study, we showed that APC7 markedly promotes antiviral innate immunity and represses the replication of RNA viruses, including Enterovirus 71 (EV71) and vesicular stomatitis virus (VSV). The study revealed that APC7 enhances the expression of IFN-I through mediating the phosphorylation and nuclear translocation of IRF3. Mechanistically, APC7 interacted with MAVS on mitochondria and promoted K63-linked ubiquitination as well as prion-like aggregation of MAVS after Sendai virus (SeV) infection. Thus, our findings identify a novel regulator of RLR-MAVS-IFN-I signaling and provide a new perspective for the treatment of RNA virus-related diseases.

## 2 Materials and methods

### 2.1 Cell culture

Human embryonic kidney cell (HEK293T), human cervical cancer cell (HeLa), and human leukemic monocyte cell (THP-1) were purchased from American Type Culture Collection (ATCC) (Manassas, VA, USA). HEK293T and HeLa cells were cultured in DMEM medium supplemented with 10% fetal bovine serum (FBS) and 1% penicillin-streptomycin. THP-1 cells were grown in RPMI 1640 medium containing 1% penicillin-streptomycin and 10% FBS. THP-1 cells were differentiated into macrophages with 100 nM 12-O-tetradecanoyl-phorbol-13-acetate (TPA) (Sigma-Aldrich; St. Louis, MO, USA) for 24 h. All cells were maintained at 37 °C in a 5% CO_2_ incubator.

### 2.2 Viruses and infections

The EV71 (Xiangyang-Hubei-09 strain, GenBank accession number JN230523.1) was obtained as previously described (29). The SeV and VSV strains were obtained from the China Center for Type Culture Collection (CCTCC) (Wuhan, China). A recombinant VSV strain expressing GFP (VSV-GFP) was kindly provided by Prof. M. Chen of Wuhan University (Wuhan, China). For viral infection assay, cells were seeded in plates and infected with the virus at the indicated multiplicity of infection (MOI) for 2 h. Subsequently, the cells were rinsed with PBS to remove unabsorbed virus and cultured in serum-free medium for the indicated time points.

### 2.3 Antibodies and reagents

Rabbit anti-EV71 VP1 (GTX132338) antibody was purchased from GeneTex (Irvine, CA, USA). Mouse anti-APC7 (sc-365649), anti-MAVS (sc-166583), and anti-ubiquitin (sc-8017) antibodies were obtained from Santa Cruz Biotechnology (Santa Cruz, CA, USA). Rabbit anti-phospho-TBK1 (5483), anti-TBK1 (3504), anti-IRF3 (11904), and anti-Lamin B1 (13435) antibodies were purchased from Cell Signaling Technology (Danvers, MA, USA). Rabbit anti-phospho-IRF3 (AB76493) antibody was obtained from Abcam (Cambridge, MA, USA). Mouse anti-Myc (60003-2-Ig), anti-GAPDH (60004-1-Ig), anti-GFP (66002-1-Ig) antibodies, and rabbit anti-HA (51064-2-AP) and anti-COXIV (11242-1-AP) antibodies were purchased from ProteinTech Group (Wuhan, China). Mouse anti-Flag (F1804) antibody was acquired from Sigma-Aldrich. Mouse anti-HA (901514) antibody was acquired from BioLegend (San Diego, CA, USA). Rabbit anti-K63-linkage ubiquitin antibody was purchased from Abways (Shanghai, China). Rabbit anti-SeV (PD029) antibody was obtained from MBL (Nagoya, Japan). Rabbit anti-Cyclin B1 (S-1407-224) antibody was obtained from Starter (Shanghai, China). The anti-GFP immunomagnetic beads were purchased from Yeasen (Shanghai, China). The double-stranded RNA (dsRNA) analog poly(I:C) (tlrl-pic) was obtained from InvivoGen (San Diego, CA, USA). MitoTracker (M22425) was purchased from Invitrogen (Carlsbad, CA, USA). The APC/C inhibitor, proTAME, was obtained from MedChemExpress (Monmouth Junction, NJ, USA).

Small interfering RNAs (siRNAs) targeting the negative control (NC) or human APC7 were designed and synthesized by GenePharma (Shanghai, China). The siRNAs (50 nM) were transfected into cells with Lipo2000 (Invitrogen) according to the manufacturer’s instructions. The sequences of all siRNAs are as follows: siNC, 5’-UUCUCCGAACGUGUCACGUTT-3’, siAPC7, 5’-GGCUGUCACAGCUUCUAUATT-3’.

### 2.4 RNA extraction and quantitative PCR

Total RNA was extracted with a Super FastPure Cell RNA Isolation Kit (Vazyme; Nanjing, China) and reverse-transcribed into cDNA using HiScript II Q Select RT SuperMix (Vazyme). Real-time quantitative PCR (qPCR) analysis was performed with SYBR qPCR Master Mix (Vazyme) on the ABI 7500 Fast Real-time PCR system (Applied Biosystems; Carlsbad, CA, USA). The relative expression changes of mRNA were calculated by the 2^-ΔΔCt^ method. The sequences of the primers used for qPCR are as follows: GAPDH, forward, 5’-AAGGCTGTGGGCAAGG-3’, reverse, 5’-TGGAGGAGTGGGTGTCG-3’; IFN-β, forward, 5’-GAGGCTTGAATACTGCCTCAA-3’, reverse, 5’-TCCTTGGCCTTCAGGTAATGCAGA-3’; ISG56, forward, 5’-GCCATTTTCTTTGCTTCCCCTA-3’, reverse, 5’-TGCCCTTTTGTAGCCTCCTTG-3’.

### 2.5 Co-immunoprecipitation (Co-IP) and immunoblot analysis

Whole-cell lysates were prepared by lysing cells in RIPA buffer (Beyotime; Shanghai, China) supplemented with a protease inhibitor cocktail (Roche; Basel, Switzerland). After centrifugation at 12,000 rpm for 10 min in a 4 °C centrifuge, a part of the lysate was reserved for immunoblot analysis, and the rest was incubated with the indicated antibodies overnight with rotation, followed by incubation with Protein A/G agarose beads (Santa Cruz) for 2 h at 4 °C. The beads were washed 4 times with RIPA buffer and denatured with SDS-PAGE loading buffer after the last wash. For immunoblot analysis, cells were lysed with RIPA buffer, and the protein concentrations were quantified using the BCA Protein Assay Kit (Solarbio; Beijing, China). Cell lysates or immunoprecipitates were loaded and separated on 10% SDS-PAGE gels, then transferred to nitrocellulose (NC) membranes (Millipore; Billerica, MA, USA). After being blocked with 5% skim milk, the membranes were probed with specific primary antibodies and corresponding secondary antibodies. Finally, protein bands were imaged on the Amersham Imager 600 (GE Healthcare; Chicago, IL, USA).

### 2.6 Semi-denaturing detergent agarose gel electrophoresis (SDD-AGE)

SDD-AGE analysis was performed as previously described (15). In brief, cells were lysed in buffer A (10 mM Tris-HCl, pH 7.5; 10 mM KCl; 1.5 mM MgCl_2_; and 0.25 M D-mannitol) supplemented with a protease inhibitor cocktail for 30 min at 4 °C. After centrifugation at 700 g for 10 min at 4 °C, the supernatants were further centrifuged at 10,000 g for 30 min at 4 °C to obtain intact crude mitochondria. The crude mitochondria (P5) were then lysed in 1 × sample buffer (0.5 × TBE, 10% glycerol, 2% SDS, and 0.0025% bromophenol blue) and loaded onto a vertical 1.5% agarose gel containing 0.1% SDS. After electrophoresis in the running buffer (0.5 × TBE with 0.1% SDS) for 1 h with a constant voltage of 100 V at 4 °C, the proteins were transferred to NC membranes for further immunoblot analysis.

### 2.7 Mitochondria isolation

Mitochondria from cells were isolated with a Mitochondria Isolation Kit (Beyotime). First, the cells were resuspended in mitochondrial isolation buffer and homogenized with a Dounce homogenizer. Then, the cell homogenates were centrifuged at 600 g for 10 min at 4 °C, and the supernatants were collected for further centrifugation at 11,000 g for 10 min at 4 °C to pellet the mitochondria. After an additional centrifugation at 12,000 g for 10 min at 4 °C, the supernatants from last step were saved as the cytosolic fraction. The mitochondrial pellets were lysed in mitochondrial lysis buffer for the following Co-IP and immunoblot analyses.

### 2.8 Luciferase assay

HEK293T cells were plated in 24-well plates and cotransfected with the IFN-β firefly luciferase reporter plasmid (100 ng), pRL-TK Renilla luciferase reporter plasmid (10 ng), empty vector or APC7 expression plasmid (200 ng), and the indicated RLR signaling-activated mutant expression plasmids (200 ng) for 24 h. Luciferase activities were determined by a Dual Luciferase Reporter Assay Kit (Vazyme) and analyzed with a luminometer (Promega; Madison, WI, USA). Firefly luciferase activity was normalized based to Renilla luciferase activity, and the data are presented as relative luciferase activity.

### 2.9 Cytoplasmic and nuclear proteins separation

The cytoplasmic and nuclear proteins were separated using a Nucleoprotein Extraction Kit (Solarbio) according to the manufacturer’s instructions. First, the cells were lysed in cytoplasmic protein extraction buffer and vortexed for 15 s. The lysates were centrifuged at 12,000 g for 10 min at 4 °C, and the supernatants were collected for cytoplasmic protein detection. Subsequently, the pellets were lysed with nuclear protein extraction buffer and vortexed for 15 s. The samples were centrifuged again at 12,000 g for 10 min at 4 °C, and the supernatants were collected for nuclear protein detection.

### 2.10 Confocal microscopy

The cells were seeded in 20-mm coverslips and transfected with the indicated plasmids for 24 h. After staining with MitoTracker for 30 min at 37 °C, the cells were fixed with 4% paraformaldehyde, permeabilized with 0.4% Triton X-100, blocked with 5% bovine serum albumin (BSA), incubated with primary antibodies, and then stained with corresponding fluorescein-conjugated secondary antibodies. Nuclei were stained with DAPI (Roche). The samples were scanned with confocal laser scanning microscopy (Leica Microsystems; Wetzlar, Germany).

### 2.11 Lentivirus packaging and stable cell line construction

The sequences of short-hairpin RNAs (shRNAs) targeting APC7 or NC were designed as follows: shNC, 5’-CAACAAGATGAAGAGCACCAA-3’; shAPC7, 5’-GGTGACAACTCAAGAGCAATC-3’. For lentivirus production, HEK293T cells were transfected with psPAX2, pMD2.G, and pLKO.1 containing shNC or shAPC7 at a ratio of 3:1:4. Lentivirus was harvested from the HEK293T cells culture medium supernatant at 24 h and 48 h post-transfection. THP-1 or HEK293T cells were infected with lentivirus for 48 h and then selected with 2.5 μg/ml puromycin (Sigma-Aldrich). The knockdown efficiency of shRNA was detected by Western blotting.

### 2.12 Statistical analysis

All experiments were reproducible and repeated at least three times. The results were presented as mean ± SD. Statistical analysis was performed using Prism 8 software (GraphPad Software Inc; San Diego, CA, USA) by unpaired Student’s *t*-test. *P*-value < 0.05 was considered statistically significant.

## 3 Results

### 3.1 APC7 inhibits RNA virus infection

**Figure 1:**
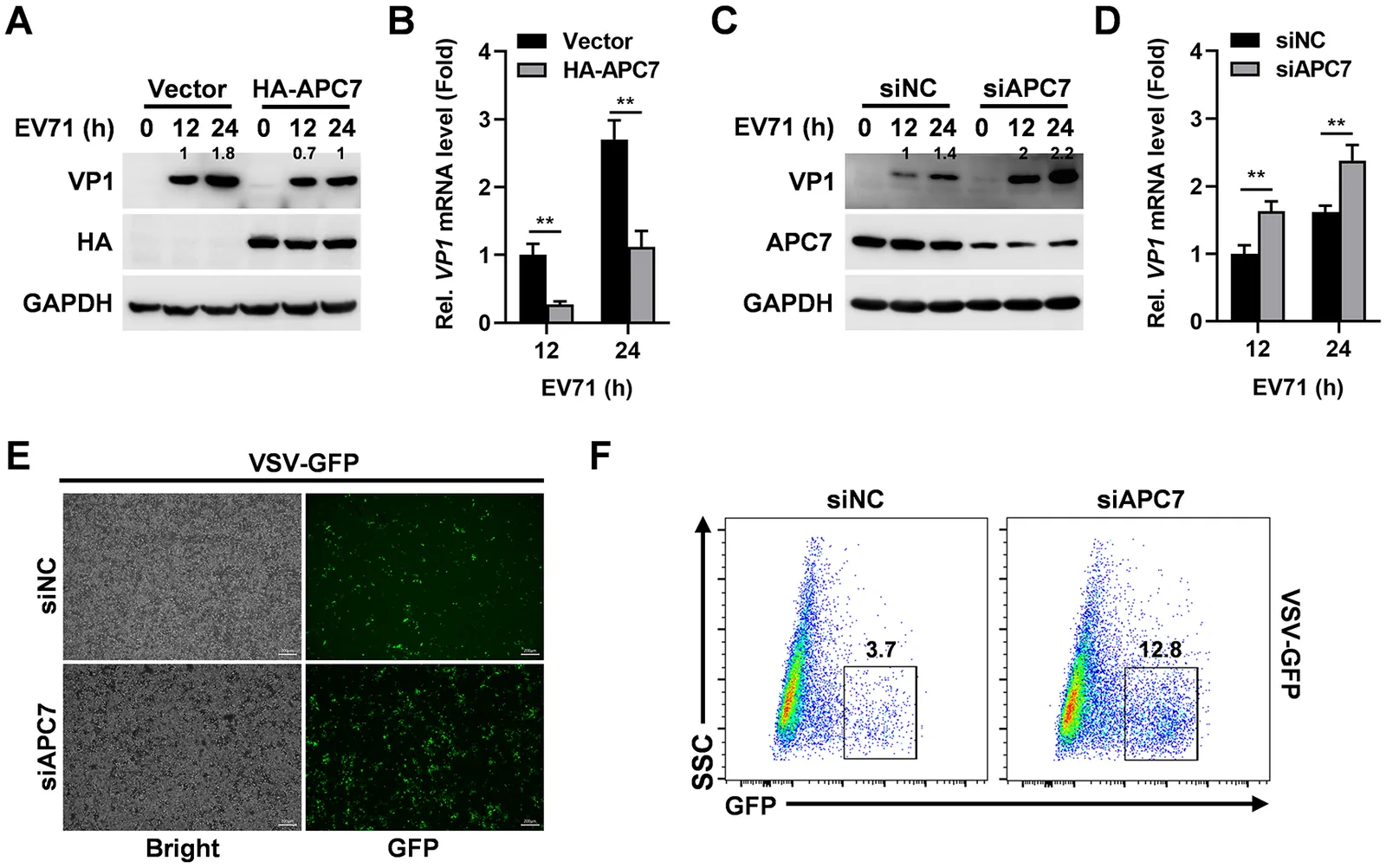
APC7 inhibits RNA virus infection. **(A, B)** HeLa cells were plated in 6-well plates and transfected with 2 μg empty vector or HA-APC7 expression plasmids; after 24 h, the cells were infected with EV71 (MOI = 1) for 0, 12, and 24 h. The expression levels of VP1, HA-APC7, and GAPDH were detected by Western blot analysis, and the level of VP1 relative to GAPDH was quantified by ImageJ **(A)**. VP1 RNA levels were determined by qPCR, and the data are presented as fold induction relative to those in empty vector-transfected cells at 12 h postinfection **(B)**. **(C, D)** HeLa cells were transfected with siNC or siAPC7 and then infected with EV71 (MOI = 1) for 0, 12, and 24 h. The levels of VP1, APC7, and GAPDH were analyzed by Western blotting, and the level of VP1 relative to GAPDH was quantified by ImageJ **(C)**. VP1 RNA levels were detected by qPCR, and the data are presented as fold induction relative to those in siNC-transfected cells at 12 h postinfection **(D)**. **(E, F)** HeLa cells were transfected with siNC or siAPC and then infected with VSV-GFP (MOI = 1) for 8 h. GFP fluorescence was imaged by fluorescence microscope **(E)** and quantified by flow cytometry **(F)**. Bar = 200 μm. The graphs are presented as mean ± SD. **P < 0.01.

To explore the antiviral potential of APC7, we first investigated the influence of APC7 on RNA virus replication. We transfected HeLa cells with an empty vector or a HA-APC7 plasmid and found that ectopic expression of APC7 significantly reduced VP1 protein and RNA levels (**Figures 1A, B**). The function of APC7 in EV71 infection was further determined using APC7-targeting siRNA and shRNA, which effectively attenuated APC7 expression (**Supplementary Figures 1A, B**). After reducing the expression of APC7, the VP1 protein level of EV71 increased (**Figure 1C**). Consistently, VP1 RNA was higher in cells transfected with siAPC7 (**Figure 1D**). We also measured the effect of APC7 on another RNA virus, VSV-GFP. Fluorescence imaging and flow cytometry results showed that GFP intensity was enhanced in APC7-knockdown cells compared to control cells (**Figures 1E, F**). Overall, these results revealed that APC7 suppresses RNA virus replication.

### 3.2 Overexpression of APC7 promotes RNA virus-induced innate immune response

**Figure 2:**
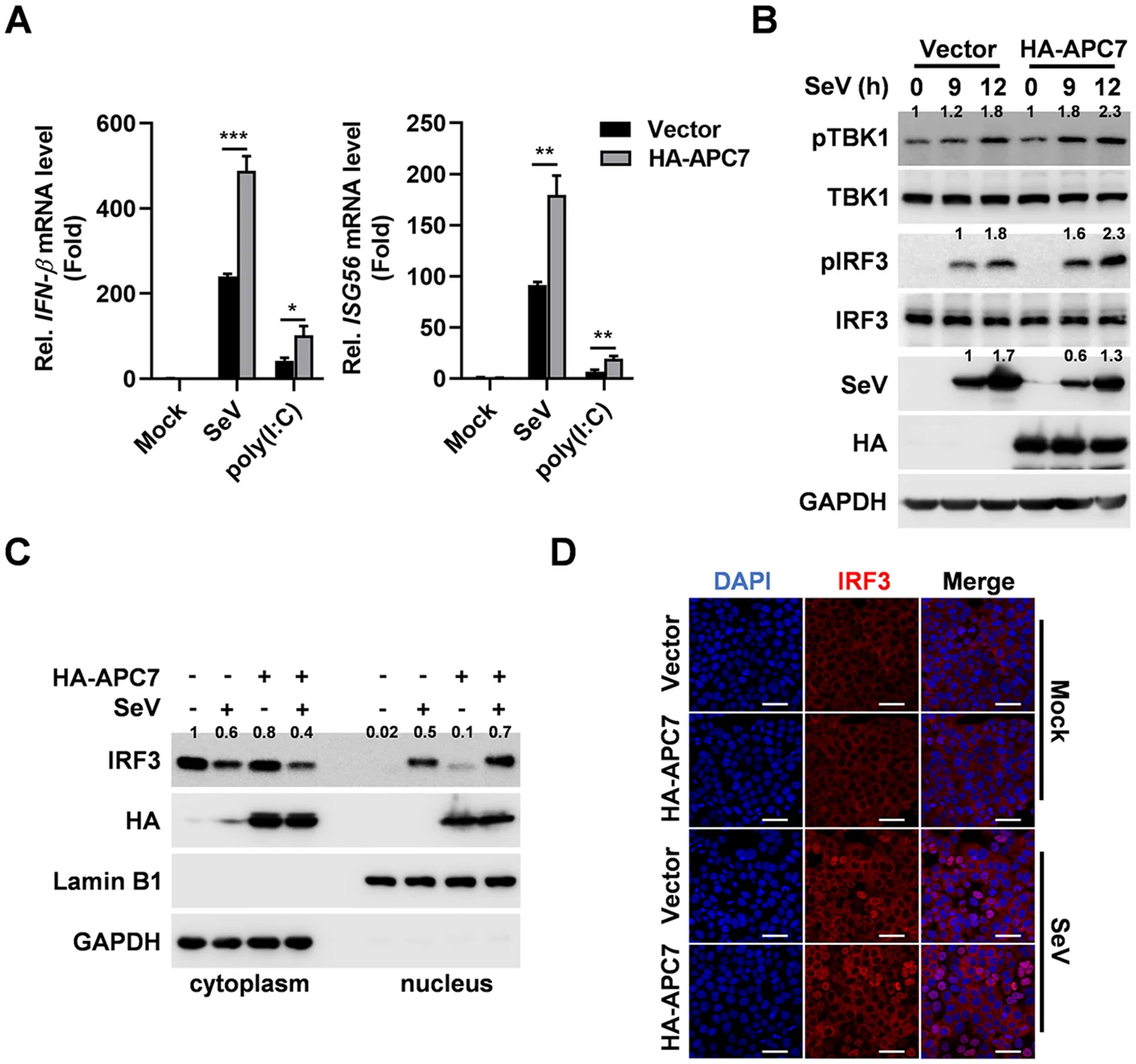
Overexpression of APC7 promotes RNA virus-induced innate immune response. **(A)** HeLa cells were transfected with empty vector or HA-APC7 plasmids for 24 h and infected with SeV (MOI = 1) or transfected with poly(I:C) (3 μg/ml) for another 16 h. The mRNA levels of IFN-β and ISG56 were determined by qPCR. **(B)** HeLa cells were transfected with empty vector or HA-APC7 plasmids for 24 h and then infected with SeV (MOI = 1) for 0, 9, and 12 h. The expression of various proteins in the cell lysates was analyzed with the indicated antibodies, and the relative levels of pTBK1/TBK1, pIRF3/IRF3 and SeV/GAPDH were quantified by ImageJ. **(C, D)** HeLa cells were transfected with empty vector or HA-APC7 plasmids for 24 h and then infected with SeV (MOI = 1) for 12 h. The cells were lysed to prepare cytoplasmic and nuclear fractions, and the levels of various proteins were determined by Western blotting **(C)**. The levels of IRF3 relative to GAPDH or Lamin B1 were quantified by ImageJ. The expression of IRF3 (red) in the nuclei (blue) was detected by confocal microscopy **(D)**. Bar = 50 μm. The graphs are presented as mean ± SD. *P < 0.05, **P < 0.01, ***P < 0.001.

Given that virus infection triggers an innate immune response, we aimed to determine whether the function of APC7 in suppressing RNA virus replication is associated with the interferon signaling pathway. HeLa cells were transfected with an APC7 expression plasmid prior to SeV infection or poly(I:C) transfection. The results demonstrated that overexpression of APC7 significantly facilitated the expression of IFN-β and ISG56 mRNA (**Figure 2A**). Furthermore, APC7 potentiated SeV-induced phosphorylation of TBK1 and IRF3 in HeLa cells (**Figure 2B**). Since phosphorylated IRF3 translocated from the cytoplasm into the nucleus to induce IFN-β transcription, the effect of APC7 on IRF3 nuclear accumulation was evaluated. Cytoplasmic and nuclear fractionation and immunofluorescence analyses collectively indicated that SeV infection promoted IRF3 translocation into the nucleus; meanwhile, overexpression of APC7 further increased nuclear accumulation of IRF3 (**Figures 2C, D**). Immunofluorescence staining also validated APC7 expression and SeV infection (**Supplementary Figure 2A**). Taken together, these data illustrated that overexpression of APC7 promotes antiviral innate immunity.

### 3.3 Knockdown of APC7 reduces RNA virus-triggered innate immune response

**Figure 3:**
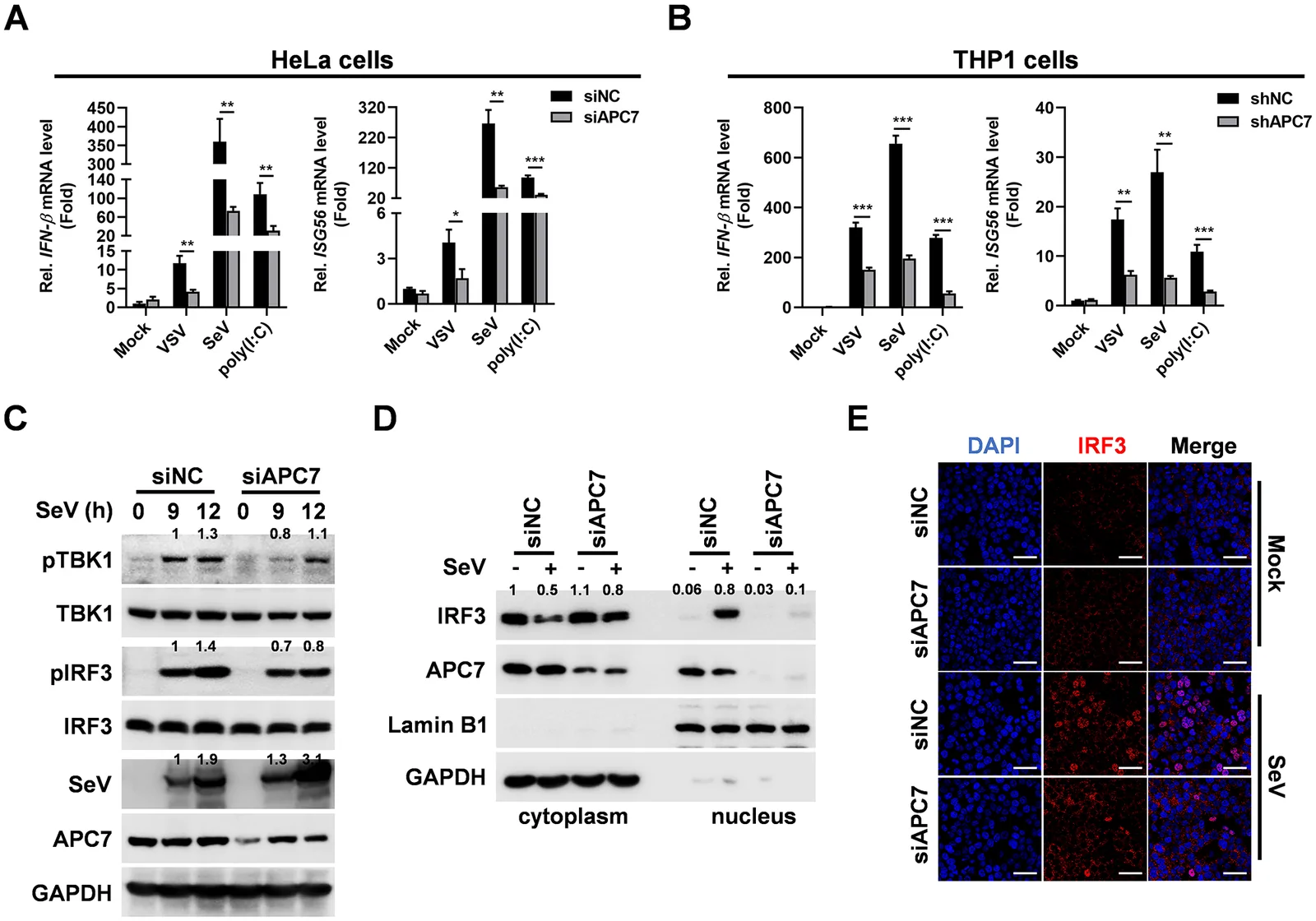
Knockdown of APC7 reduces RNA virus-triggered innate immune response. **(A)** HeLa cells were transfected with siNC or siAPC7 for 24 h and then infected with VSV or SeV at an MOI of 1, or transfected with poly(I:C) (3 μg/ml) for 16 h. The mRNA levels of IFN-β and ISG56 were determined by qPCR. **(B)** THP-1 cells stably expressing shNC or shAPC7 were generated and infected with VSV or SeV at an MOI of 1, or transfected with poly(I:C) (3 μg/ml) for 12 h. The mRNA levels of IFN-β and ISG56 were determined by qPCR. **(C)** HeLa cells were transfected with siNC or siAPC7 for 24 h and then infected with SeV (MOI = 1) for the indicated time. The levels of various proteins in the cell lysates were detected by Western blot analysis; the relative expression levels of pTBK1/TBK1, pIRF3/IRF3 and SeV/GAPDH were quantified by ImageJ. **(D, E)** HeLa cells were transfected with siNC or siAPC7 for 24 h and then infected with SeV (MOI = 1) for 12 h. The cells were lysed to prepare cytoplasmic and nuclear fractions, and the levels of various proteins were determined by immunoblotting **(D)**. The levels of IRF3 relative to GAPDH or Lamin B1 were quantified by ImageJ. The expression of IRF3 (red) in the nuclei (blue) was detected by confocal microscopy **(E)**. Bar = 50 μm. The graphs are presented as mean ± SD. *P < 0.05, **P < 0.01, ***P < 0.001.

Next, we determined the role of APC7 in the innate immune response by knockdown of APC7. After transfection with siNC or siAPC7, HeLa cells were infected with VSV or SeV, or treated with poly(I:C). Compared to cells transfected with siNC, the mRNA levels of IFN-β and ISG56 were reduced in cells transfected with siAPC7 (**Figure 3A**). Meanwhile, THP-1 cells stably expressing shNC or shAPC7 were constructed, and we found that silencing APC7 reduced the mRNA levels of IFN-β and ISG56 upregulated by VSV, SeV or poly(I:C) (**Figure 3B**). The levels of phosphorylated TBK1 and IRF3 activated by SeV or VSV infection were reduced in cells expressing siAPC7 or shAPC7 (**Figure 3C**; **Supplementary Figure 2B**). Consistently, subcellular fractionation and immunofluorescence analyses indicated that knockdown of APC7 attenuated SeV-induced nuclear accumulation of IRF3 (**Figures 3D, E**; **Supplementary Figure 2C**). Furthermore, we investigated whether the function of APC7 in innate immunity depended on APC/C activity. HeLa cells were treated with various concentrations of the APC/C inhibitor proTAME. The results showed that proTAME significantly stabilized the classical APC/C substrate Cyclin B1 (**Supplementary Figure 3A**), confirming its effective inhibition of APC/C. However, APC/C inactivation by proTAME did not affect SeV-activated IRF3 phosphorylation (**Supplementary Figure 3B**). Altogether, these findings suggested that APC7 plays an essential role in the innate immune response triggered by RNA viruses.

### 3.4 APC7 targets MAVS in the RLR signaling pathway

**Figure 4:**
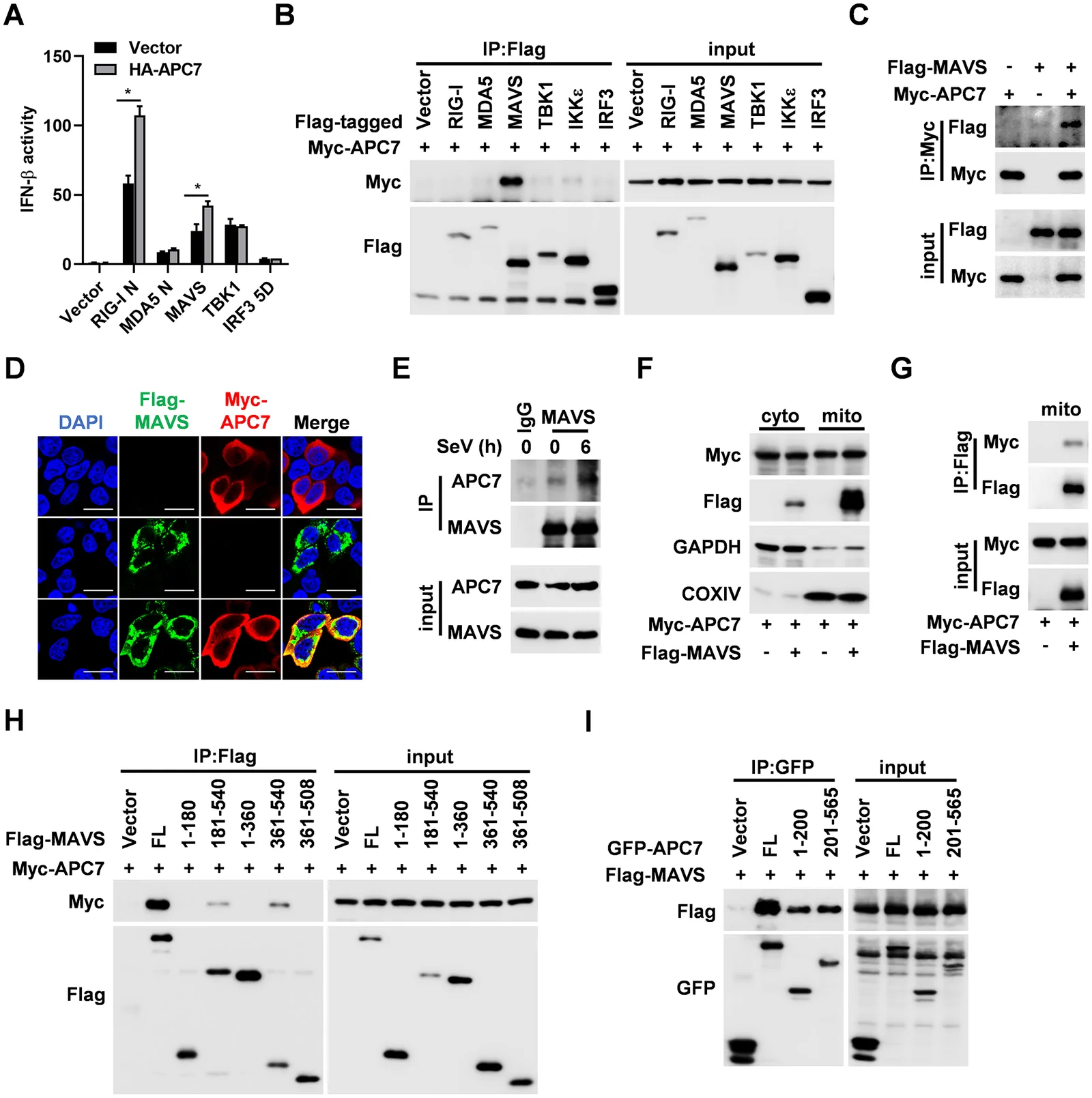
APC7 targets MAVS in the RLR signaling pathway. **(A)** HEK293T cells were seeded in 24-well plates and transfected with pRL-TK, IFN-β luciferase, empty vector or HA-APC7, along with RIG-I N, MDA5 N, MAVS, TBK1, or IRF3 5D plasmids as indicated. IFN-β activity was measured by luciferase assay. **(B)** HEK293T cells were cotransfected with Myc-APC7 and Flag-RIG-I, Flag-MDA5, Flag-MAVS, Flag-TBK1, Flag-IKKϵ, or Flag-IRF3 plasmids. The cell lysates were prepared and immunoprecipitated with an anti-Flag antibody; the expression of Myc-APC7 and Flag-tagged proteins was detected by immunoblotting. **(C)** HEK293T cells were transfected with Myc-APC7 and Flag-MAVS plasmids and immunoprecipitated with an anti-Myc antibody. The IP complex and input samples were analyzed by Western blotting with antibodies against Myc and Flag. **(D)** HeLa cells were transfected with plasmids as indicated for 24 h; the cells were fixed for immunofluorescence analysis. The locations of Myc-APC7 (red), Flag-MAVS (green), and nuclei (blue) were imaged by confocal microscopy. Bar = 20 μm. **(E)** HEK293T cells were uninfected or infected with SeV (MOI = 1) for 6 h; the cells were then lysed and immunoprecipitated with IgG or an anti-MAVS antibody. The expression of APC7 and MAVS in IP and input samples was measured with APC7 and MAVS antibodies. **(F, G)** HeLa cells were plated in 10 cm dishes and transfected with Myc-APC7 alone or Myc-APC7 and Flag-MAVS together. The cells were collected for mitochondrial isolation, and the mitochondria were lysed for immunoblotting and Co-IP analysis. The expression of Myc-APC7, Flag-MAVS, GAPDH, and COXIV in the cytosol and mitochondria was determined by Western blot **(F)**. The levels of Myc-APC7 and Flag-MAVS in IP and input samples were detected by immunoblotting **(G)**. **(H)** HEK293T cells were transfected with Myc-APC7 and full-length or truncated Flag-MAVS plasmids, and then immunoprecipitated with an anti-Flag antibody. The IP and input samples were immunoblotted with the indicated antibodies. **(I)** HEK293T cells were transfected with Flag-MAVS and full-length or truncated GFP-APC7 plasmids and immunoprecipitated with anti-GFP immunomagnetic beads. The IP and input samples were immunoblotted with the indicated antibodies. The graphs are presented as mean ± SD. *P < 0.05. Cyto, cytosol; mito, mitochondria; FL, full length.

To understand the mechanism by which APC7 regulates RNA virus-induced innate immunity, we cotransfected the HA-APC7 plasmid with different RLR pathway-activating mutants. Our results revealed that APC7 enhanced IFN-β promoter activity driven by RIG-I N and MAVS, but not by MDA5 N, TBK1, or IRF3 5D (**Figure 4A**), indicating that APC7 acts at the level of MAVS and its upstream signaling. Next, HEK293T cells were transfected with Myc-APC7 and Flag-tagged RLR pathway protein expression plasmids. Co-IP results showed that only Myc-APC7 expression was detectable in the Flag-MAVS-IP complex (**Figure 4B**), which demonstrates that MAVS specifically interacts with APC7. Reciprocal Co-IP experiments verified the interaction between APC7 and MAVS (**Figure 4C**). Immunofluorescence analysis illustrated that APC7 and MAVS were colocalized in the cytoplasm (**Figure 4D**). Endogenous interaction assays demonstrated the interaction between APC7 and MAVS, which was enhanced upon SeV infection (**Figure 4E**). Next, we examined whether APC7 interacts with MAVS on mitochondria. First, Flag-MAVS and Myc-APC7 expression plasmids were transfected into HeLa cells. Then, the mitochondrial and cytosolic components were fractionated. Immunoblot analysis demonstrated that MAVS was mainly localized on mitochondria (**Figure 4F**), consistent with the previous research findings (30, 31). Additionally, APC7 was also present on mitochondria (**Figure 4F**). Subsequently, the isolated mitochondrial proteins were subjected to immunoprecipitation, and the results demonstrated that there was a significant interaction between APC7 and MAVS on mitochondria (**Figure 4G**). Furthermore, the domains responsible for the APC7-MAVS interaction were explored. We observed that APC7 co-precipitated with MAVS fragment 361–540 aa rather than 1–360 aa or 361–508 aa (**Figure 4H**), suggesting that the transmembrane domain (508–540 aa) is essential for the interaction. In addition, we found that both the N-terminus and C-terminus of APC7 were necessary for MAVS binding (**Figure 4I**). Collectively, these observations demonstrated the interaction between APC7 and MAVS.

### 3.5 APC7 enhances the aggregation of MAVS

**Figure 5:**
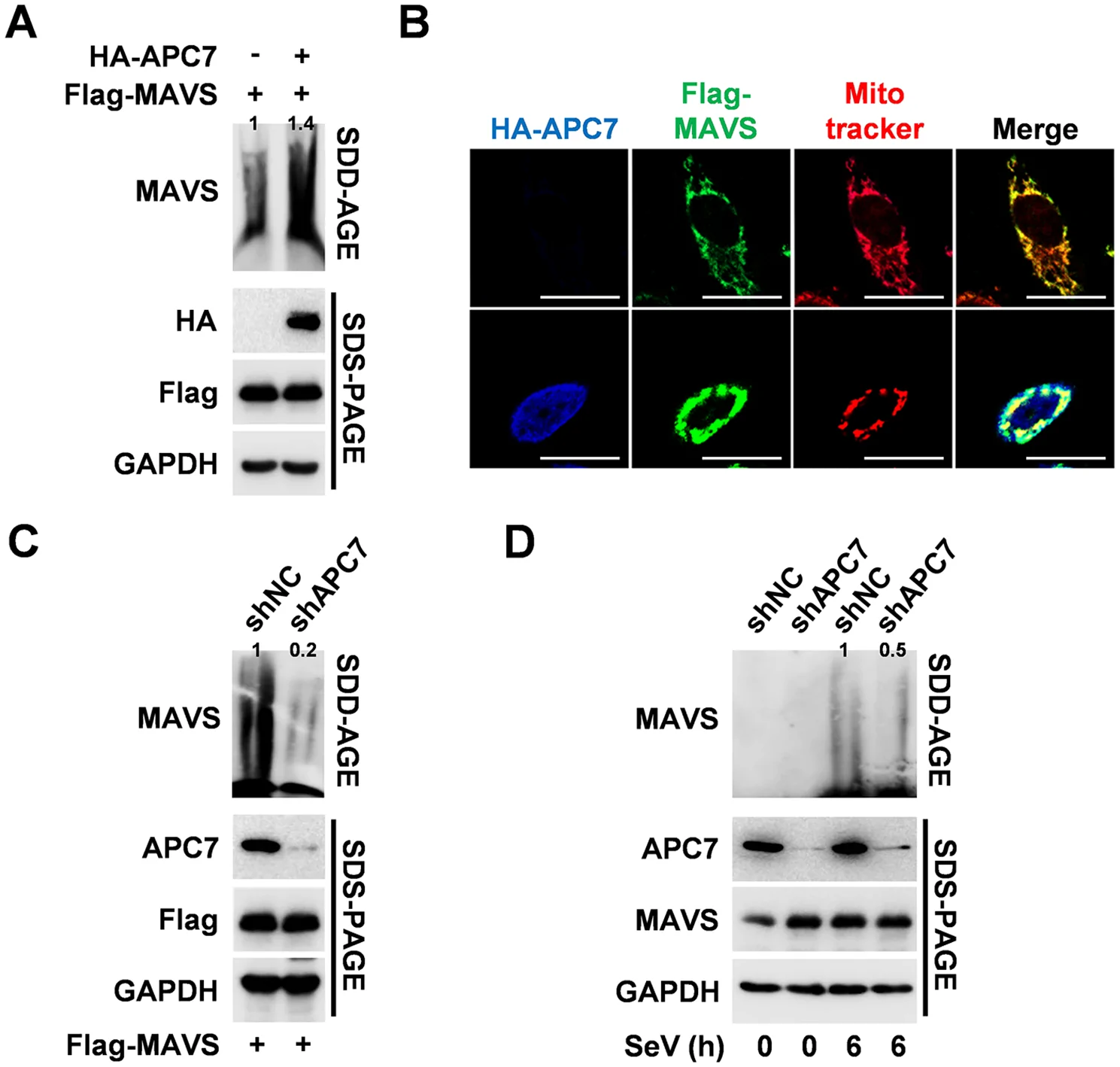
APC7 enhances the aggregation of MAVS. **(A)** HEK293T cells were transfected with HA-APC7 and Flag-MAVS expression plasmids. MAVS aggregates were detected by SDD-AGE; the expression of HA-APC7, Flag-MAVS, and GAPDH was determined by SDS-PAGE. The level of MAVS aggregation relative to total MAVS was quantified by ImageJ. **(B)** HeLa cells were transfected with HA-APC7 and Flag-MAVS plasmids. The cells were stained with anti-HA (blue) and anti-Flag (green) antibodies and MitoTracker (red) for confocal microscopy. Bar = 20 μm. **(C)** HEK293T cells stably expressing shNC or shAPC7 were transfected with Flag-MAVS plasmid. MAVS aggregates were detected by SDD-AGE; the expression of APC7, Flag-MAVS, and GAPDH was determined by SDS-PAGE. The level of MAVS aggregation relative to total MAVS was quantified by ImageJ. **(D)** THP-1 cells stably expressing shNC or shAPC7 were infected with SeV (MOI = 1) for 6 h. MAVS aggregates were detected by SDD-AGE, and the expression of APC7, MAVS, and GAPDH was determined by SDS-PAGE. The level of MAVS aggregation relative to total MAVS was quantified by ImageJ.

As an important adaptor protein in the innate immune response, MAVS forms prion-like aggregates on mitochondrial membranes after being activated by upstream signals (5). Therefore, we next investigated whether APC7 affects the formation of MAVS aggregates. HEK293T cells were transfected with HA-APC7 and Flag-MAVS plasmids, followed by mitochondrial isolation and SDD-AGE analysis to detect MAVS aggregates. Overexpression of APC7 enhanced the aggregation level of MAVS (**Figure 5A**). Immunofluorescence analysis also revealed that APC7 promoted MAVS aggregation (**Figure 5B**). The effect of APC7 knockdown on MAVS aggregate formation was then investigated. HEK293T cells stably expressing shNC or shAPC7 were transfected with a Flag-MAVS expression plasmid and subjected to SDD-AGE analysis. We found that knockdown of APC7 reduced the aggregation of MAVS (**Figure 5C**). Meanwhile, THP-1 cells stably expressing shNC or shAPC7 were infected with SeV. The results showed that knockdown of APC7 diminished MAVS aggregation induced by SeV infection (**Figure 5D**). Therefore, our data suggested that APC7 promotes the formation of MAVS aggregates.

### 3.6 APC7 facilitates the K63-linked polyubiquitination of MAVS

**Figure 6:**
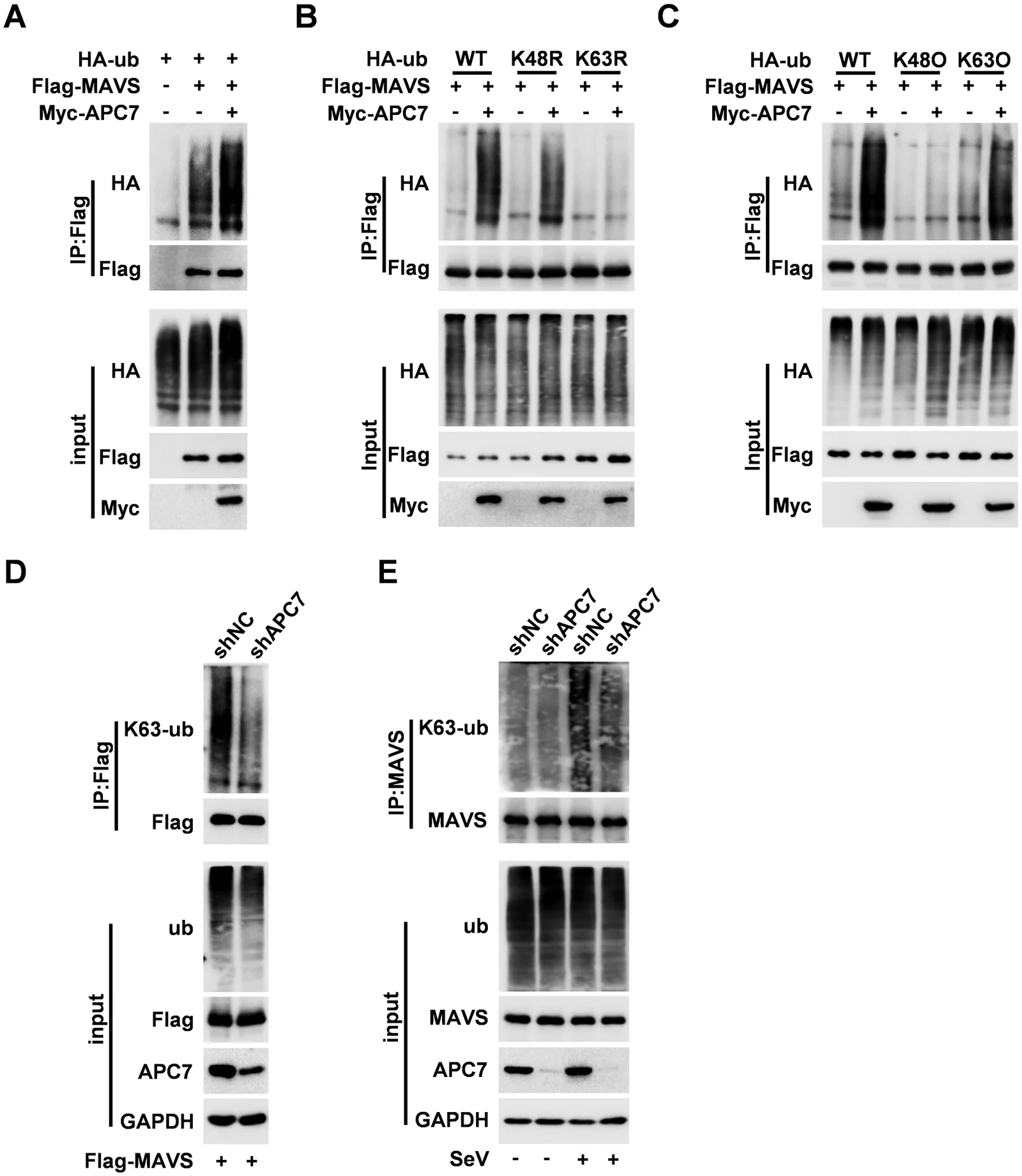
APC7 facilitates the K63-linked polyubiquitination of MAVS. **(A)** HEK293T cells were transfected with HA-ub, Flag-MAVS, and Myc-APC7 expression plasmids for 24 h; the cell lysates were then immunoprecipitated with an anti-Flag antibody. The expression of HA-ub and Flag-MAVS in the IP complex, as well as HA-ub, Flag-MAVS, and Myc-APC7 in input samples, was determined by immunoblotting. **(B, C)** HEK293T cells were transfected with expression plasmids for Flag-MAVS, Myc-APC7, and either HA-ub, HA-K48R, HA-K63R, HA-K48O, or HA-K63O. 24 h later, the cell lysates were prepared and immunoprecipitated with an anti-Flag antibody. The IP and input complexes were analyzed with the indicated antibodies. **(D)** HEK293T cells stably expressing shNC or shAPC7 were transfected with Flag-MAVS plasmid and immunoprecipitated with an anti-Flag antibody. The expression of K63-linked ubiquitin and Flag-MAVS in IP samples, as well as ubiquitin, Flag-MAVS, APC7, and GAPDH in input samples was detected by immunoblotting. **(E)** THP-1 cells stably expressing shNC or shAPC7 were infected with SeV (MOI = 1) for 6 h and immunoprecipitated with an anti-MAVS antibody. The expression of K63-linked ubiquitin and MAVS in IP samples, as well as ubiquitin, MAVS, APC7, and GAPDH in input samples was detected by immunoblotting. Ub, ubiquitin.

As previously reported, ubiquitination is essential for regulating MAVS activity and its aggregation on mitochondria (10). With this in mind, we next evaluated whether APC7 regulates MAVS ubiquitination. We found that overexpression of APC7 increased the ubiquitination level of MAVS (**Figure 6A**). In order to study the type of APC7-enhanced ubiquitination, HEK293T cells were transfected with plasmids expressing Flag-MAVS, Myc-APC7, and either wild-type ubiquitin or different ubiquitin mutants, including those with a single lysine residue mutated (K48R, K63R) or with only one lysine residue retained (K48O, K63O). The results suggested that overexpression of APC7 promoted the K63-linked rather than K48-linked polyubiquitination of MAVS (**Figures 6B, C**). Furthermore, HEK293T cells stably expressing shNC or shAPC7 were transfected with Flag-MAVS plasmid, and Co-IP assays demonstrated that knockdown of APC7 inhibited K63-linked ubiquitination of MAVS (**Figure 6D**). Consistently, our endogenous ubiquitination experiments showed that K63-linked ubiquitination of MAVS was significantly reduced in APC7-knockdown THP-1 cells after SeV infection (**Figure 6E**). These findings collectively indicated that APC7 facilitates K63-linked polyubiquitination of MAVS.

## 4 Discussion

RNA virus infections cause a variety of diseases, including influenza, pneumonia, autonomic dysfunction, poliomyelitis, aseptic meningitis, and hemorrhagic fever, posing a serious threat to public health (32–34). Innate immunity constitutes the primary host barrier against pathogen invasion. It can rapidly recognize viral infection through PRRs, activate signaling cascades, and produce IFN-I and proinflammatory factors, ultimately inhibiting viral replication and inducing the death of infected cells (1, 8). Here, we identified a previously undefined role of APC7 in inhibiting RNA virus replication, which is independent of its cell-cycle function. Mechanistically, APC7 facilitated the antiviral innate immune response by targeting MAVS. Furthermore, APC7 promoted K63-linked polyubiquitination and prion-like aggregation of MAVS. These investigations demonstrated that APC7 may serve as a positive regulator of innate immunity and highlight its potential as a target for antiviral therapy.

APC/C is a sophisticated molecular complex that participates in multiple signaling pathways and cellular processes through subunit interaction and substrate degradation (23). Consequently, any alterations in the APC/C subunits or coactivators may compromise or abolish its activity, thereby triggering a cascade of downstream responses. Increasing attentions are being paid to the independent roles of APC/C subunits (35–37). Several studies have reported the distinct function of APC/C subunits or coactivators in viral infection and innate immunity; for example, APC10 regulates NLRP3 inflammasome activation during the cell cycle in a manner independent of APC/C (38), and the APC/C substrate adaptor Cdh1 facilitates antiviral innate immunity by regulating MAVS-TRAF3/6 signaling (39). However, the function of APC7 in viral infection or the innate immune response remains unclear. In this study, our initial results indicated that APC7 inhibited the replication of EV71 and VSV and enhanced the phosphorylation of TBK1 and IRF3 induced by RNA virus infection or poly(I:C) transfection, as well as the subsequent nuclear translocation of IRF3, ultimately triggering the production of IFN-I. These findings suggested that APC7 exerts an antiviral effect through the activation of innate immunity. Notably, we did not observe any significant alteration in APC7 expression in response to EV71, SeV, or VSV infections. Therefore, we hypothesize that the role of APC7 in innate immunity may not depend on its inducible upregulation. Instead, APC7 might function as a constitutively available regulator that is constantly poised to modulate MAVS-mediated signaling, independent of viral stimulation. Furthermore, we demonstrated that the APC/C inhibitor proTAME had no effect on the antiviral response, indicating that the function of APC7 in innate immune regulation may not involve APC/C catalytic activity.

As a crucial adaptor of the RLR signaling pathway, MAVS contains a C-terminal transmembrane domain that anchors it to the mitochondrial outer membrane (30). Our data indicated that APC7 interacted with MAVS and further confirmed that APC7 and MAVS were colocalized on mitochondria. Besides, we found that APC7 interacted with the transmembrane domain of MAVS, suggesting that APC7 and MAVS form a complex on the mitochondrial outer membrane to regulate downstream signaling. Previous studies have shown that upon RNA virus infection, MAVS forms prion-like aggregates to transmit activation signals (5, 40). Here, we revealed that APC7 further enhanced SeV-induced MAVS aggregation, indicating that APC7 facilitates the innate immune response against RNA virus by promoting MAVS aggregation. Moreover, APC7 promoted K63-linked polyubiquitination of MAVS after RNA virus infection. Given that APC7 lacks intrinsic E3 ligase activity and its effect on innate immune regulation is proTAME-insensitive, our findings imply a non-canonical role for APC7 outside the APC/C. We propose two principal, non-mutually exclusive models to account for these observations. First, APC7 may function as a scaffold that brings specific E3 ubiquitin ligases into proximity with MAVS. Second, APC7 might act as a competitive inhibitor of deubiquitinases, sequestering negative regulators away from MAVS. Although TRIM31 has been reported as a MAVS-targeting ligase, we did not detect a stable APC7-TRIM31 interaction (data not shown). In consideration of these unresolved possibilities, a mass spectrometry-based screening for APC7-interacting partners under both basal and virus-infected conditions is warranted in future studies.

There are some limitations in our study. First, most overexpression and mechanistic mapping experiments were performed in HEK293T and HeLa cells, which are not representative of the immune microenvironment. In addition, the function and mechanism of APC7 in innate immunity have not yet been validated *in vivo*. Finally, the precise mechanism by which APC7 regulates MAVS aggregation formation and K63-linked polyubiquitination remains to be elucidated. These important questions could be addressed in future studies.

In summary, the present study identified that a subunit of APC/C, APC7, is a novel regulator of innate antiviral immunity. The research revealed that APC7 interacts with MAVS on mitochondria and enhances its subsequent aggregation and K63-linked polyubiquitination, thereby promoting the production of IFN-I and inhibiting the replication of RNA viruses. Collectively, this study contributes to a deeper understanding of the innate immune regulatory network and provides a novel perspective for further investigations on the functions of APC/C subunits.

## Data Availability

The original contributions presented in the study are included in the article/**Supplementary Material**. Further inquiries can be directed to the corresponding author.
