# Supplementary material for: The APC/C subunit APC7 exerts antiviral effects by targeting the adaptor protein MAVS
Source: Front Immunol. 2026 Aug 11;17:1912055. doi: 10.3389/fimmu.2026.1912055 (PMC13504260; doi:10.3389/fimmu.2026.1912055)
Supplement: Supplementary file 1 [file DataSheet1.docx]

Supplementary Material

**Supplementary Figure 1**

**Supplementary Figure 1. The knockdown efficiency of siRNA or shRNA specific to APC7**

(**A**) HeLa cells were transfected with siNC or siAPC7, and the expression of APC7 and GAPDH was analyzed by Western blotting. (**B**) THP-1 cells stably expressing shNC or shAPC7 were generated, and the knockdown efficiency of APC7 was evaluated by immunoblotting.

**Supplementary Figure 2**

**Supplementary Figure 2. Knockdown of APC7 inhibits the antiviral immune response**

(**A**) HeLa cells were transfected with HA-APC7 expression plasmid for 24 h and then infected with SeV (MOI = 1) for 12 h. SeV (green), HA-APC7 (red), and nuclei (blue) were visualized by confocal microscopy. Bar = 50 μm. (**B**) THP-1 cells stably expressing shNC or shAPC7 were infected with VSV (MOI = 1) for 0, 4, and 8 h. The expression of various proteins in the cell lysates was analyzed with the indicated antibodies, and the relative levels of pTBK1/TBK1 and pIRF3/IRF3 were quantified by ImageJ. (**C**) THP-1 cells stably expressing shNC or shAPC7 were infected with SeV (MOI = 1) for 8 h. The expression of IRF3 (red) in nuclei (blue) was detected by confocal microscopy. Bar = 50 μm.

**Supplementary Figure 3**

**Supplementary Figure 3. APC7 regulates antiviral innate immunity independent of APC/C activity**

(**A**) HeLa cells were treated with proTAME (0, 10, 20, and 50 μM) for 12 h. The expression of Cyclin B1 and GAPDH was determined by immunoblotting, and the level of Cyclin B1 relative to GAPDH was quantified by ImageJ. (**B**) HeLa cells were treated with proTAME (50 μM) for 12 h and then infected with SeV (MOI = 1) for 12 h. The levels of pIRF3, IRF3, and GAPDH were determined by Western blot analysis, and the level of pIRF3 relative to IRF3 was quantified by ImageJ.
